# Kahneman’s Insights for Climate Risks: Lessons from Bounded Rationality, Heuristics and Biases

**DOI:** 10.1007/s10640-025-00980-4

**Published:** 2025-03-14

**Authors:** W. J. Wouter Botzen, Louison D. Thepaut, Sanchayan Banerjee

**Affiliations:** 1 Institute for Environmental Studies (IVM), Vrije Universiteit Amsterdam, Amsterdam, Netherlands; 2 School for Government, The Policy Institute, King’s College London, London, UK

**Keywords:** Bounded rationality, Climate change risks, Heuristics, Kahneman, Natural disaster

## Abstract

Daniel Kahneman’s pioneering research in behavioral economics has profoundly influenced the field of environmental economics, shaping what is now known as behavioral-environmental economics. This paper provides a scoping review of how Kahneman’s theories have been applied by environmental economists to individual decision-making for climate change risks. We focus on deviations from rational behavior that impact climate adaptation decisions, such as loss aversion, the underweighting of low-probability events and the influence of heuristic-driven System 1 thinking over analytical System 2 reasoning. Our review outlines diversity in methodologies, including household surveys and economic experiments, used to analyze actions like investments in climate resilience and the purchase of disaster insurance. We synthesize these findings showing how Kahneman’s legacy explains suboptimal preparedness behaviors and discuss policy strategies derived from these insights, such as risk communication, nudges, and financial incentives for disaster preparedness. We conclude by proposing an agenda for future research to more systematically assess Kahneman’s ideas across various climate risk contexts and to deepen the application of Kahneman’s theories in tackling broader, wicked environmental problems that require changing human behaviors.

## Introduction

Daniel Kahneman’s groundbreaking research at the intersection of psychology and (behavioral) economics has profoundly impacted the field of environmental economics, leading many early scholars to establish a new line of research now known as “behavioral-environmental economics” (Shogren and Taylor 2008). Given environmental economics’ focus on understanding individual preferences for nature, the environment, and sustainable behaviors, it is unsurprising that environmental economists have increasingly adopted behavioral economic theories as the descriptive backbone of their work (Croson and Treich 2014; Kesternich et al. 2017). Kahneman’s refreshing ideas about limits to human decision-making ability, such as bounded rationality as well as the heuristics and biases paradigm, triggered research to overcome limitations to neo-classical economics. This research informed the design of behavioral-environmental policy instruments that encourage sustainable consumption (e.g. Carlsson et al., 2021; Schubert 2017). Testing deviations from the rational choice model, as inspired by Kahneman, is critical for effective environmental policy design. Identifying when and why these systematic patterns of deviation occur helps create behavioral policy interventions that can guide behavior toward societally beneficial actions. This is especially relevant in environmental economics research concerning individual decisions about climate risks, which are often associated with systematic behavioral biases (e.g., Kunreuther and Botzen 2022).

A growing number of studies have explored decision-making in the context of climate change risks, aiming to better understand individual choices related to adaptation and mitigation (Kuhlicke et al. 2023). Researchers in this field have typically employed household surveys assessing actual, intended, or preferred climate adaptation actions, as well as economic laboratory experiments in controlled settings (Robinson and Botzen 2019). These methods have been used to examine investments in climate loss mitigation measures (e.g., Mol et al. 2024) and the purchase of insurance to cover climate-related risks (e.g., Kunreuther and Michel-Kerjan 2015). Promoting adaptation actions is crucial, as natural disaster losses are rising rapidly worldwide (Hoeppe 2016). Climate change is expected to accelerate this trend by increasing the frequency and severity of extreme events like severe floods, storms, and droughts (IPCC 2021). While public sector adaptation policies play a role in limiting these losses, individual actions, such as making properties resilient to extreme weather and purchasing disaster insurance, are also essential (Botzen 2021).

Despite the cost-effectiveness of certain climate adaptation measures, like subsidized flood insurance in the United States, many individuals still fail to adopt them (Atreya et al. 2015). This can be attributed to deviations from the standard rational economic choice model, as highlighted by Kahneman’s research in various contexts. Examples include the underweighting of low-probability natural disaster risks, as explained by Kahneman and Tversky’s (1979) Prospect Theory, and the influence of dual-process theories of decision-making, such as Kahneman’s System 1 and System 2 thinking (Kahneman 2011). Intuitive System 1 thinking, based on heuristics, often prevails over the more deliberate System 2 thinking when dealing with climate change risks, which are generally unfamiliar to decision-makers (Kunreuther 2020). Heuristics that contribute to inadequate disaster preparedness include inertia related to the status quo and the underestimation of low-probability risks in the absence of disasters, influenced by availability bias (Tversky and Kahneman 1974; Kahneman et al. 1991).

The seminal work on heuristics and biases paradigm of decision-making by Kahneman initially had its origins in other applications than climate change risks, and often involved generically framed choices (Tversky and Kahneman 1974; Kahneman and Tversky 1979). This inspired environmental economists to design empirical studies to analyze if similar deviations from rational behavior are observed in a context that represents climate change-related risks. Understanding this behavior, for instance regarding the taking of climate adaptation measures, can subsequently inform the design of policies that mitigate the potential impacts of climate change. Examples include risk communication strategies to overcome the underestimation of climate change risk in the absence of natural disasters (Bradt 2022), nudges that work with the status quo bias to make having protection measures the standard (Robinson et al. 2021), and financial incentives that address suboptimal disaster preparedness due to boundedly rational behavior (Kunreuther 2021).

In recognition of Kahneman’s substantial contributions to environmental economics, this paper provides a scoping review^1^ of studies on individual decision-making related to climate change risks, inspired by his legacy. The review aims to gather evidence on whether the systematic deviations from the rational choice model, as identified by Kahneman, also apply to this context. Additionally, it explores how Kahneman’s theories have influenced this field and discusses the policy implications for mitigating climate change risks. Finally, we offer recommendations for future research that builds on the decision theory foundations established by Kahneman.

The rest of the paper is structured as follows. Section 2 describes the decision-making theories and heuristics by Kahneman, which have been used as inspiration for the behavioral studies focused on climate change-related risks. In particular, Sect. 2.1 focusses on Kahneman’s work on heuristics and biases, Sect. 2.2 on Prospect Theory and other related decision theories, Sect. 2.3 on experienced utility and Sect. 2.4 on dual process theory. In line with our objective, Sect. 3 reviews applications of these theories and ideas from Kahneman to individual decision-making related to climate change risks and their evidence, following a similar order of subsections. Section 4 discusses these key findings in Sect. 4.1 and their policy implications in Sect. 4.2. Section 5 concludes with an agenda for future research.

## Kahneman’s Pioneering Work on Bounded Rationality, Heuristics and Biases

### Kahneman’s Work on Heuristics and Biases

The heuristics-and-biases research program, pioneered by Tversky and Kahneman in the 1970s, offers a foundational model for understanding decision-making and judgment under uncertainty. As the name suggests, it rests on two core concepts: (1) individuals use mental shortcuts, or “heuristics,” when navigating probabilistic decisions, and (2) these heuristics can result in consistent and systematic deviations from rational behavior, referred to as “biases.” Together, this framework highlights our reliance on quick-thinking rules when making various decisions and underscores the significant role cognitive biases play in shaping our choices under uncertainty.

Heuristics-and-biases is a prominent psychological paradigm often contrasted with the Simple Heuristics approach developed by Gerd Gigerenzer. The Simple Heuristics perspective emphasizes that heuristics are adaptive and efficient tools for managing uncertainty and need not be seen as leading to systematic failures. According to this perspective, when heuristics yield poor outcomes, the solution lies in refining our heuristics rather than abandoning them. Kahneman and Tversky illustrate the heuristics-and-biases paradigm with a distance analogy: just as our sense of vision, while generally reliable, can be a misleading (biased) measure of distance, heuristics often lead to irrational and statistically inconsistent decision-making. In their influential 1974 *Science* paper, they describe three key heuristics that frequently cause such deviations.

The first heuristic, “representativeness”, addresses how people judge the probability of one event based on how similar it is to another. For example, a bland description of a person might lead us to think they are likely a librarian, even if, statistically, that is improbable. Kahneman and Tversky’s experiments showed that the representativeness heuristic gives rise to biases due to insensitivity to (1) the prior probability of outcomes (a failure to apply Bayes’ rule), (2) sample size effects, and (3) predictability, as well as misconceptions about (4) chance, and (5) regression to the mean. The gambler’s fallacy, a well-known cognitive bias, exemplifies this: people mistakenly believe that a random sequence, like a series of coin tosses, is self-correcting. For instance, after a series of red outcomes on a roulette wheel, they may expect black to be more likely, even though each spin is independent. Terrell’s (1994) study on betting behaviors in lottery games demonstrates this bias in a financial context, showing that bettors were less likely to bet on numbers that had recently won. The tendency to move to any global representation from a local sequence as the later expands is simply an artefact of dilution of deviations and not necessarily chance corrections as falsely assumed.

The second shortcut, the “availability heuristic”, pertains to how people judge the likelihood of an event based on how easily examples come to mind. Salient or familiar information can disproportionately influence our sense of probability, as was demonstrated by the influential work of Slovic et al. (1977) and Schoenmaker et al. (1979). Through a series of experiments, Kahneman and Tversky showed that the availability heuristic produces various biases linked to the prominence of certain memories, the salience of one’s mental search processes, limited imagination, or the formation of illusory correlations (Kahneman and Tversky 1996; Tversky and Kahneman 1974).

The third heuristic involves “anchoring and adjustment”. Here, individuals make estimates by starting from an initial value (an anchor) and adjusting from there. However, these adjustments are often insufficient, leading to inaccurate judgments. People tend to rely too heavily on the anchor, even when it is irrelevant to the task at hand.

The heuristics-and-biases research program expanded significantly following Tversky and Kahneman’s groundbreaking 1974 work. They, along with other scholars, made many influential contributions, such as the 1984 exploration of how framing effects influence decision-making. They demonstrated that people tend to be risk-averse when considering gains but risk-seeking when facing potential losses. This behavior highlights the tendency to overweight sure outcomes relative to those with moderate probabilities. Importantly, Kahneman and Tversky discovered that the biases identified within the heuristics-and-biases framework are pervasive across diverse populations, suggesting that statistical inconsistencies are not limited to specific subgroups but are, in fact, a widespread feature of human cognition (Kahneman and Tversky 1974).

While most of the heuristics-and-biases research program developed generally under Kahneman and Tversky, the findings were quickly applicable to specific decision-making domains, such as for risky decisions relating to climate change. For example, research indicates that people can over(under)-weigh their own climate risk perceptions based on their salience of such events which can consequently affect how they respond to such risks, for example, by investing more (less) in insurance against such risks (Kunreuther and Botzen 2022). For example, experiencing a climate related disaster may, according to the availability heuristic, imply that individuals find it easier to imagine that such a disaster will happen again and demand protection against this risk. Although, the opposite could also happen if people do not view such events as independent and act according to the Gambler’s fallacy. Reliance on such heuristics combined with our intuitive cost-benefit balancing can also explain demographic differences in how precautionary informed risk assessments are in the context of climate risks (Sunstein 2006). The heuristics-and-biases research program also generates implications for climate policymaking, in turn, for if people are likely more responsive to information that is salient, framing policies to match their own priors or subjective beliefs is more likely to be persuasive as well as will garner more public support (Nelson et al. 1997).

### Prospect Theory and Other Related Decision Theories

The invention of Prospect Theory is one of the major advances that Kahneman together with his colleague Tversky made in the literature about decision making under risk (Kahneman and Tversky 1979).^2^ The motivation behind developing Prospect Theory as an alternative descriptive model of choice can be found in the observation by Kahneman and Tversky of pervasive inconsistencies with standard Expected Utility. These observed deviations from rational choice include loss aversion in which equivalent losses loom larger in decisions than equivalent gains, and the overweighting of low probabilities in decisions. This choice behavior deviates from a concave utility function over final monetary wealth amounts and the linear processing of probabilities in Expected Utility Theory. Instead, Kahneman and Tversky (1979) propose to capture loss aversion through a utility function in Prospect Theory in which a utility value is assigned to monetary gains and losses relative to some neutral reference point. Moreover, this utility function is concave for gains and convex for losses, which are multiplied by a loss aversion factor. Moreover, in calculating the expected utility value the probabilities of outcomes are replaced by decision weights (Kahneman and Tversky 1979). These weights are derived from a probability weighting function that generally overweighs low probabilities and underweights high probabilities. In a later version, Tversky and Kahneman (1992) further developed Prospect Theory with cumulative decision weights, which can be applied to both decisions under risk and uncertainty. This version is called Cumulative Prospect Theory and allows for different probability weighting to occur in the gain and loss domains (Tversky and Kahneman 1992).

An implication of Prospect Theory is that risk aversion is determined by the combination of both utility curvature and probability weighting, while in Expected Utility Theory, risk aversion is only due to a concave utility function over final wealth (Kahneman and Tversky 1979). The utility function of Prospect Theory implies risk aversion in the gains domain and risk seeking behavior in the domain of losses. However, overweighting of low-probability losses may imply that individuals are risk averse for these losses and hence purchase insurance, while underweighting of high-probability losses can imply risk seeking behavior for those stakes (Tversky and Kahneman 1992). Similarly overweighting of low-probability gains may explain risk-seeking behavior in the gain domain and, therefore, gambling (Tversky and Kahneman 1992). Another implication of Prospect Theory is that shifts in the reference point may influence risk preferences. For instance, incomplete adaptation to recent losses can induce risk seeking behavior in some contexts to offset the recent loss (Kahneman and Tversky 1979).

Kahneman and Tversky (1979) recognized that the probability weighting function is not well defined near the end points, which includes low probabilities near zero. This can apply to low-probability high-impact climate risks, such as flood or storm risks that may be over- or underweighted in specific contexts. Hence it is of interest to empirically examine probability weighting in various climate contexts and how this influences individual decisions with regards to climate adaptation or mitigation actions. Other interesting climate research may involve examining how loss aversion influences individual preferences for actions that mitigate climate risk, and how potential changing reference points after incurring climate change-related losses influence risk preferences.

### Experienced Utility

An influential line of research by Kahneman is his concept of ‘experienced utility’ (Kahneman et al. 1997; Kahneman 2000). This notion relates to viewing utility as experiences of pain and pleasure. Kahneman and Krueger (2006) described various measures for directly measuring experienced utility, such as using self-reported happiness scales. He discussed a range of evidence showing that these scales logically related to individual characteristics, circumstances, and life events, such as health, income employment. One of the major conclusions drawn by Kahneman and Kreuger (2006) from this literature on self-reported happiness is that it can have a profound impact on economics, for instance, because it allows for a more direct measurement of welfare effects. Kahneman and Sugden (2005) discuss both the opportunities and pitfalls for using subjective reported happiness as a policy evaluation tool since it may overcome some of the limitations of stated-preference studies. For instance, if self-reported happiness is a reliable indicator of well-being, then researchers could use it to examine how external factors, such as pollution, influence happiness. Next, they can quantify the monetary equivalent of these effects based on the relationship between income and happiness. Kahneman and Sugden (2005) illustrate this approach for deriving large causal effects, such as the impact of living near an airport, but they note that deriving causal effects on well-being may be more challenging for small-scale effects. Linking this approach to climate change research can be relevant as the impacts of climate change, such as natural disaster events, are likely to have significant effects on wellbeing. This opens the door for research that uses the life-satisfaction approach to examine the impact of climate-related natural disasters and mitigation factors (e.g., adaptation measures) on well-being. Such empirical studies could provide the foundation for monetarizing these relationships, offering input for cost-benefit analyses of climate policy.

### Dual Process Theory: Systems 1 and 2

Dual process theories broadly refer to a figurative partition in human cognitive process relating to how information is processed and how behavioral outcomes are generated in response to that. Much of the earlier work on dual process theories was conceptualized in the form of two families (Kahneman and Frederick 2005) or systems (Stanovich and West 2000) or types (Evans 2007) of human cognition: “intuition”, which is a quick response, often thought to be an associative cue, potentially with evolutionary origins; and “reason”, which is an effortful and deliberative response, and kicking in to correct quick responses, as and when needed. The foundation of these distinct thinking pathways in psychology were widely popularized through Daniel Kahneman’s (2011) book, *Thinking*,* Fast and Slow* which conveyed a simple message: the fast, intuitive system uses mental shortcuts, conserves cognitive resources, and generates quick responses whereas the slower, more deliberate system, in contrast, demands greater mental effort and produces responses over a longer time, known as reflective or slow cognition. Together, these cognitive systems can interact to help humans process and respond to the various cues they encounter. It should be noted that several studies underlying the argumentation in Kahneman’s (2011) book have come under scrutiny as part of the ‘replication crisis’ in psychology and have been criticized for relying on too small samples (Schimmack et al. 2017). Kahneman (2017) agreed with this criticism and indicated he ‘placed too much faith in underpowered studies”. Nevertheless, given the objective of our study, we believe it is relevant to review applications of dual process theory due to the important influence it has on the research field.

While most of this work has been developed independently and under various names (for an overview, see Banerjee and John 2024), the early development of dual process theory can be traced to various works by Daniel Kahneman and colleagues. For example, Kahneman’s capacity model of attention (1973) explains attentional resource limitations by proposing that attention is a finite and flexible resource that is allocated based on the demands of tasks. Kahneman uses the analogy of an electrical grid: performing a demanding task is like plugging in an appliance, which draws electricity from the system. If too many appliances (or tasks) require power, the grid becomes overloaded, and the system fails. This model underscores the limitations and automatic nature of how cognitive resources are distributed based on varying task requirements. Since the brain’s capacity for mental effort is limited, effortful processes (System 2) interfere with each other, while effortless processes (System 1) do not significantly disrupt or suffer from interference when combined with other tasks. Based on this criterion, many different functions can be assigned to be designated as either System 1 or 2.

This elegant yet simple formulation of Kahneman’s insights to dual process theories can help with numerous deductions for environmental decisions made in the context of climate change. For example, when thinking about climate risks, people might respond with instinctive emotions (System 1), such as fear or apathy. If climate change feels abstract or distant in time and space, people may be less motivated to act (Spence et al. 2012). Current research has shown that building on these instincts by enabling people to visualize the future better can lead to improved adaptation responses as shown in the case of investment in risk reducing measures in the context of flooding (Mol et al., 2022) or forest conservation (Banerjee and Ferreira 2024). Similarly, when faced with climate investment decisions, humans often need to consider and process information, all of which can be significantly resource intensive. Kahneman’s insights from capacity-models, further combined with recent work in behavioral science on boosting people capacities (Hertwig 2017), has been shown to significantly improve demand for flood insurance (Bradt 2022).

## How Kahneman Inspired Environmental Economics Research on Decision-Making Under Climate Change Risks

### Heuristics and Biases

Kahneman’s work on heuristics and biases reveals how cognitive shortcuts shape individual responses to environmental risks, often leading to suboptimal decisions. This section explores four key heuristics and biases—availability heuristic, representativeness heuristic, confirmation bias, and the outrage effect—demonstrating their influence on risk perception, behavior, and policy preferences in the context of climate change and environmental risks.

#### Availability Heuristic: The Impact of Recent, Salient Events on Risk Perception

The availability heuristic, as described by Kahneman and Tversky, suggests that people estimate the likelihood of an event based on how easily they can recall similar past occurrences. In environmental economics, this heuristic explains why personal experiences with floods, heatwaves, and other extreme weather events amplify individuals’ perceptions of climate risk. For example, Lohmann and Kontoleon (2023) demonstrate that personal experiences with floods and heatwaves in England and Wales heightened perceptions of climate change risk, especially in areas close to flood events, which highlights the role of proximity in risk salience. The study found that while such experiences increased climate risk perception, they did not consistently lead to long-term pro-environmental behavior. This suggests that while recent, intense events make climate risks more vivid, their impact on sustained behavior change is limited.

Similarly, Ji and Cobourn (2020) reveal that farmers in Idaho rely heavily on recent weather events, particularly drought conditions, to shape their expectations for future climate conditions. Rather than basing their decisions on long-term climate averages, by focusing on short-term water availability and temperature fluctuations, farmers adjusted crop choices and acreage in ways that led to cyclical over- and under-corrections, reflecting a short-term focus that often disrupts long-term planning. This drought-focused study highlights the impact of availability bias in agricultural decision-making, where reliance on recent drought conditions can lead to suboptimal outcomes in water-scarce regions.

Additional studies illustrate how the availability heuristic influences risk perceptions across various contexts. Wolf and Takeuchi (2022) investigate the effects of the Gokayama Dam in Japan on property values after Typhoon Prapiroon. They found that following the typhoon, property values in flood-protected areas rose, as individuals overestimated the flood prevention capacity of the dam based on the vivid memory of the recent disaster. Similarly, McCoy and Walsh (2018) show that housing prices in wildfire-prone areas in Colorado declined after wildfires but recovered within a few years as the memory of the event faded. This temporary increase in perceived wildfire risk reflects the salience of recent, vivid events and the short-lived nature of availability bias.

Gallagher (2014) investigates flood insurance take-up in the U.S., showing that people in areas recently affected by floods were more likely to purchase insurance. However, this effect diminished over time as memories of the event faded, underscoring the transitory nature of availability-driven risk perceptions. Hennighausen and Suter (2020) also document a significant decrease in property prices in flooded areas of Boulder County, Colorado, immediately after a major flood, reflecting heightened risk perceptions. Yet, over time, property prices rebounded as the availability of the flood in people’s memories faded.

These findings underscore the potential for policymakers to leverage heightened awareness generated by recent extreme weather events to promote climate adaptation and mitigation measures. Localized communication strategies could be particularly effective following such events, as proximity and recency amplify climate risk perceptions. By aligning communication with the availability heuristic, policymakers can foster sustainable responses, especially in areas frequently affected by climate-related disasters.

#### Representativeness Heuristic: Overweighting Recent Events in Catastrophe Risk

While the availability heuristic focuses on the ease of recalling past events, the representativeness heuristic refers to the tendency to judge probabilities based on how typical or representative recent events are of future outcomes. There is some overlap between the two, as both relate to recent experiences, but they differ in their cognitive mechanism. The availability heuristic is about ease of recall, while the representativeness heuristic is about judging similarity to past patterns.

For example, Volkman-Wise (2015) applies the representativeness heuristic to catastrophe insurance, showing that individuals are more likely to invest in insurance immediately after a disaster due to an overestimated likelihood of recurrence. This behavior leads to high insurance demand post-disaster, with individuals often underweighting risk pre-disaster. The study suggests that policies ensuring consistent insurance coverage and sustained risk awareness could mitigate this bias.

Wolf and Takeuchi (2022) also explore representativeness in flood protection, showing that residents near the Gokayama Dam in Japan perceived the dam as highly effective after Typhoon Prapiroon, leading to increased property values in protected areas. The recent disaster shaped perceptions of the dam’s reliability, reflecting the tendency to overestimate future safety based on recent events.

Given these findings, policymakers should ensure that insurance and flood protection strategies account for the representativeness heuristic by maintaining a balanced view of disaster likelihood that does not overly depend on recent events. Providing consistent information about risks can help mitigate over- and under-insuring behaviors that arise due to this bias.

#### Confirmation Bias: Filtering Information to Support Climate Beliefs

Confirmation bias—the tendency to favor information that supports existing beliefs—can affect how individuals interpret and recall climate-related events. Zappalà (2023) examines this bias in Bangladesh, showing that individuals with pre-existing beliefs about increasing drought frequency were more likely to overestimate past drought events. This selective recall aligns with motivated reasoning, distorting climate information and complicating efforts to align perceptions with objective assessments.

Public communication strategies addressing confirmation bias should provide balanced information on climate risks, especially in vulnerable communities. Tailored campaigns that directly engage with existing beliefs and highlight long-term trends could help counteract selective recall, encouraging adaptive responses to climate threats (Zappalà 2023).

#### Outrage Effect: Moral Judgements on Human-Caused Environmental Damage

Kahneman’s outrage effect highlights that moral judgements can influence people’s WTP for mitigating environmental harm, especially when the harm is seen as preventable. Bulte et al. (2004) illustrate this in a study on threatened seals in the Waddenzee, showing that respondents were more willing to pay to address environmental problems caused by human activities, such as oil drilling, than by natural causes, like a virus. This moral dimension suggests that public sensitivity to human-caused harm can shape conservation efforts.

For policymakers, framing environmental policies to emphasize human responsibility could increase public support. Highlighting preventable or human-caused degradation may heighten moral urgency, enhancing support for conservation and climate initiatives.

In summary, Kahneman’s insights into heuristics and biases reveal cognitive tendencies shaping individual responses to climate risks. By understanding these biases, policymakers can design more effective communication strategies, risk management policies, and conservation campaigns that address inherent biases, fostering accurate risk perception and proactive environmental behavior.

### Prospect Theory

This section examines the application of Prospect Theory in environmental economics research, focusing on enhancing our understanding of decision-making in climate-related contexts, particularly regarding risk responses, policy design, and adaptation strategies.

#### Risk Responses and Probability Weighting: Misaligned Perceptions of Climate Risk

A key contribution of Prospect Theory is the concept of probability weighting, where people tend to overestimate small probabilities and underestimate larger ones. This bias is especially relevant in climate change contexts, where low-probability, high-impact events (e.g., extreme floods or hurricanes) drive public and policy responses. Input in the probability weighting function is how people perceive the probability. This is examined by Turner and Landry’s (2022) who study flood perceptions among U.S. homeowners. They reveal that individuals frequently misperceive flood probabilities based on subjective estimates, often without specific probability information provided. Their findings indicate that homeowners tend to overestimate the likelihood of flood events but underestimate potential damages, reflecting a cognitive bias that emphasizes the probability of experiencing a disaster over its severity. This study suggests that misperceptions arise more from individual heuristics than from systematic probability weighting applied to given data.

In their study on flood protection in Switzerland, Glatt et al. (2019) use probability weighting to analyze willingness to pay for flood protection measures. They assess how individuals respond when provided with specific probability information, finding that participants overweight the small probability of extreme flood events. This results in a higher demand for flood protection than traditional Expected Utility Theory would predict, as respondents demonstrate heightened sensitivity to low-probability, high-risk events. This behavior aligns with Kahneman’s observation that individuals do not process probabilities linearly, potentially leading to over-investment in risk protection.

However, Robinson and Botzen (2020) show that this finding about individual weighting of flood probabilities is highly context-dependent and varies across population subgroups. They estimate the probability weighting function using an economic experiment of decisions to purchase flood insurance among a sample of homeowners. These experimental participants were from the Netherlands, where flood probabilities are generally much lower than in other European countries and flood events have historically been rare. The results indicate that in this context very low flood probabilities are generally underweighted. This is empirically explained by individuals applying a threshold of concern model, which is associated with more probability underweighting when flood probabilities are low. Moreover, an internal locus of control and the anticipated regret of uninsured insured losses are associated with less probability underweighting. The study recommends improving risk communication and to bundle flood risk insurance coverage with coverage for more common higher-probability risk in within the same policy (Robinson and Botzen 2020). Implementing these recommendations increase the likelihood that individuals perceive the risk as exceeding their threshold level of concern, which can trigger overweighting of low flood probabilities.

Together, these studies highlight distinct yet related applications of Prospect Theory in climate risk: Glatt et al. (2019) and Robinson and Botzen (2020) direct applications of probability weighting suggests that low-probability risks can drive demand for protective measures when explicit probabilities are given, while Turner and Landry’s findings emphasize the role of subjective probability overestimations in shaping demand. For policymakers, these insights suggest that interventions might focus on calibrating risk communication to better align public perception with objective risk levels, especially in contexts where low probabilities are underestimated and underweighted in protection decisions.

#### Loss Aversion: Shaping Climate Disaster Preparedness and Insurance Demand

Loss aversion—the notion that losses loom larger than gains—is another cornerstone of Prospect Theory with substantial implications for climate change adaptation. This bias implies that individuals are more sensitive to potential losses than equivalent gains, particularly relevant after experiencing climate-induced losses. Page et al. (2014) study on flood-affected homeowners in Brisbane, Australia, offers a clear illustration of loss aversion in action. They found that individuals affected by the 2011 floods were significantly more likely to choose a risky lottery option over a guaranteed small payout, a behavior predicted by the “reflection effect” in Prospect Theory, where people become risk-seeking in the domain of losses. Here, affected homeowners’ decisions to pursue risky options to recover losses after the flood event exemplifies how loss experience can drive individuals towards decisions that might appear suboptimal under traditional utility-maximization perspectives.

Loss aversion also plays a critical role in the demand for climate risk insurance, as highlighted by Botzen and van den Bergh’s (2009) study on flood insurance in the Netherlands. In flood-prone regions, individuals exhibit an increased willingness to pay for insurance that mitigates potential losses, behavior exceeding predictions from standard economic models. By incorporating loss aversion into their models combined with an overweighting of low-probability flood risk according to the standard probability weighting function of Prospect Theory, the researchers demonstrate that traditional utility frameworks do not capture the public’s actual willingness to invest in insurance.

Building on this, Koetse and Brouwer (2015) examine how loss aversion affects flood risk mitigation decisions in the IJsselmeer region of the Netherlands. Their study finds a significant disparity between willingness to pay (WTP) and willingness to accept (WTA) for flood protection. Specifically, residents demanded significantly more compensation to forgo flood protection than they were willing to pay to secure it, reinforcing the idea that perceived losses weigh more heavily than equivalent gains.

Kahsay and Osberghaus (2017) further support these insights in their study on storm damage and risk perception in Germany. The study reveals that individuals who experience extreme weather events exhibit persistent increases in loss aversion, particularly when making financial decisions. The study shows that affected individuals are more likely to invest in insurance products, indicating that prior losses create a heightened sensitivity to future risk. However, they also find that risk perceptions gradually fade over time, leading to decreasing insurance uptake in the years following a disaster. This temporal decay in loss aversion suggests that while climate disasters can drive insurance demand, maintaining this demand requires continuous risk communication to reinforce the perceived value of protection.

In all cases, Kahneman’s concept of loss aversion provides a valuable framework for understanding risk-related decision-making. Policymakers aiming to increase insurance uptake or encourage disaster preparedness might leverage this insight by framing insurance as protection against significant potential losses, tapping into people’s natural aversion to losing what they value. Given that risk perceptions decline over time, policies should also focus on sustained messaging and long-term incentives to maintain preparedness beyond the immediate aftermath of extreme weather events. This approach could foster higher participation in natural disaster insurance schemes and build more resilient communities.

#### Reference Dependence and Anchoring in Environmental Risk Valuation

Reference dependence, where individuals evaluate outcomes relative to a specific baseline or “reference point,” is another essential aspect of Prospect Theory. This concept is particularly influential in understanding how people value climate adaptation efforts, as perceptions of gains and losses often hinge on reference points that shift over time or in response to changing environmental conditions.

Slunge et al. (2019) examine reference dependence in the context of tick-borne disease risks in Sweden. Here, respondents’ WTP for risk reduction varies based on whether the risk is new or already endemic. Those in areas where ticks were an emerging threat exhibited a higher WTP to avoid exposure, viewing the risk as a deviation from their reference point of safety. Conversely, in regions where tick prevalence was established, people displayed a lower WTP, indicating an adaptation to the reference point of living with elevated risk. These examples highlight how reference points strongly influence risk valuation in environmental contexts, where individuals assess the value of adaptation measures based on perceived shifts from baseline expectations.

Further supporting this perspective, Viscusi and Huber (2011) investigate reference-dependent valuations of risk and explain why willingness-to-accept (WTA) often exceeds WTP in environmental health contexts. Their study finds that individuals’ reference points significantly shape their valuation of risk reduction, with WTA exceeding WTP due to differences in how people mentally frame potential losses versus gains. Interestingly, they identify that this gap is driven more by reference dependence in monetary costs rather than in risk probabilities. This insight has direct implications for climate adaptation policies: individuals may demand disproportionately high compensation (WTA) to forgo flood protection or climate resilience measures but exhibit a lower WTP when asked to contribute proactively to those same protections.

For policymakers, reference dependence suggests that public support for climate adaptation measures may be higher if interventions are framed to maintain baseline safety standards rather than as new investments. In designing risk reduction policies, governments can tailor communications to address specific reference points held by different communities. This may involve providing information that contextualizes risks relative to local historical baselines, helping individuals understand how certain risks have evolved and why protective measures are essential.

#### Overcoming the Energy Efficiency Gap: Loss Aversion and Probability Weighting in Policy Design

Kahneman’s work on cognitive biases has also influenced research on energy efficiency, where behavioral barriers often prevent individuals from making cost-effective investments. The “energy efficiency gap” is a phenomenon where consumers avoid energy-efficient options despite their financial and environmental benefits (Gerarden et al., 2017). Heutel’s (2019) study, conducted with a sample of 2,045 U.S. individuals representing a broad cross-section of the population, provides insight into this gap by applying Prospect Theory. Through a choice experiment using lottery questions, Heutel explores how loss aversion and probability weighting shape individuals’ hesitancy to invest in energy-efficient technologies, such as alternative fuel vehicles or efficient appliances.

The study finds that US consumers’ strong aversion to losses discourages them from incurring upfront costs for energy-efficient products, as they perceive these expenses as immediate losses rather than future gains. Heutel further notes that probability weighting discourages investment, as consumers undervalue the small but certain savings from energy-efficient choices, amplifying their preference to avoid initial expenses.

This pattern has significant policy implications, suggesting that standard economic incentives may be insufficient to close the energy efficiency gap. By framing subsidies or incentives to emphasize the avoidance of future financial losses rather than merely offering upfront cost reductions, policymakers might align more effectively with consumers’ loss-averse tendencies (Heutel 2019). Additionally, policies that account for probability weighting, such as emphasizing the cumulative effect of small energy savings over time, could help shift consumer preferences towards energy-efficient investments (Heutel 2019). In this way, Kahneman’s insights provide a foundation for designing energy policies that resonate with people’s natural biases, encouraging more sustainable consumer behavior in line with climate goals.

#### Prospect Theory as a Foundation for Climate Decision-Making

In summary, Prospect Theory’s insights into probability weighting, loss aversion, and reference dependence provide a robust framework for understanding decision-making under climate risks. Probability weighting helps explain why people overestimate the likelihood of extreme events, leading to higher demand for flood protection or insurance, while loss aversion accounts for the increased willingness to invest in preventive measures after experiencing climate-related losses. Reference dependence shapes how people value adaptation efforts, as they anchor their preferences relative to baseline safety conditions. Across studies, these themes reveal how Kahneman’s contributions transcend traditional economic models, offering a more nuanced view of human behavior in uncertain environmental contexts. For policymakers, leveraging these insights involves designing climate policies that recognize and address cognitive biases in public decision-making, ultimately fostering resilience in the face of growing climate risks.

### Experienced Utility and Alternative Decision Theories

Kahneman’s groundbreaking work on experienced utility, our ability to derive well-being from actual experiences rather than purely from anticipated utility, has given environmental economists a powerful tool for understanding how people respond to climate and environmental risks. Experienced utility shifts the focus from hypothetical choices to the emotional impact of lived events, offering a more nuanced perspective on how individuals react to unpredictable environmental stresses. Moreover, using other alternative decision theories, such as Random Regret Minimization (RRM) and Rank-Dependent Utility (RDU), Kahneman’s insights highlight how decision-making under stress deviates from traditional utility-maximizing models, especially in high-stakes scenarios like climate-induced natural disasters (Chorus 2010; Quiggin 1982).

#### Life Satisfaction as a Measure of Experienced Utility During Environmental Shocks

A central contribution of Kahneman’s framework of experienced utility is its focus on life satisfaction as a measure of well-being under environmental stress, such as floods and dust storms. In their study on flood risks across 16 European countries, Luechinger and Raschky (2008) show that life satisfaction significantly declines following flood events with an estimated reduction of approximately 0.035 points on a 4-point scale. To offset this loss in well-being, they estimate a compensating surplus requirement equivalent to about 23.7% of an average household’s annual income to prevent a certain flood event. By quantifying these declines, they demonstrate that floods do not only cause economic damage but also significant subjective well-being losses, a factor often overlooked in conventional economic assessments. The authors also find that countries with broad insurance coverage experience lower drops in life satisfaction after flood events, indicating that risk transfer mechanisms like mandatory insurance can mitigate the psychological impact of natural disasters. This finding emphasizes the role of insurance in enhancing resilience to environmental shocks. The authors propose the “life satisfaction approach” as a method of non-market valuation for natural disasters, positioning it as a complement to traditional methods like hedonic pricing. Their approach broadens the view of flood impacts on human well-being, reinforcing Kahneman’s assertion that life satisfaction offers a valuable lens through which to assess experienced utility.

Similarly, Jones (2023) examines the transient effects of dust storms on life satisfaction in the United States. The study finds that life satisfaction decreases on days with dust storms, though the impact is lessened by public warnings. Jones’s research shows how real-time alerts can reduce immediate well-being losses, providing a practical policy recommendation for strengthening early warning systems. Both studies illustrate how experienced utility can quantify non-economic losses from environmental stressors, supporting a shift in climate policy towards addressing psychological as well as economic resilience. By focusing on experienced rather than hypothetical impacts, these studies affirm the importance of using well-being data to capture the true costs of climate risks on communities.

Building on this, Hudson et al. (2019) assess the impacts of flood events and household preparedness on subjective well-being in flood-prone areas of France. They find that subjective well-being is significantly reduced by flooding events, with well-being losses, equating to approximately €150,000 per household immediately following an event. Further, Hudson et al. (2019) find that measures increasing flood preparedness, such as elevating your home or adopting flood-resistant designs, increases well-being by approximately €31,000, highlighting the role that psychological resilience plays as risk preparedness reduces the subjective impacts of floods even when physical risks remain. This aligns with Kahneman’s theory, that measuring well-being offers a broader understanding on the costs of climate related risks, by incorporating psychological and emotional dimensions often overlooked in traditional economic models.

More recently, Djoumessi (2023) finds that households affected by severe floods in Tanzania experienced a 34% decline in agricultural productivity and a lasting decrease in life satisfaction. The study further shows that those receiving social transfers or living near forests were able to mitigate these negative impacts, suggesting that both social and environmental safety nets play a role in post-disaster adaptation. The findings emphasize the need to incorporate psychological factors in modelling as climate disasters can have significant long-term psychological and economic effects. Djoumessi (2023) suggests that adaptive mechanisms such as access to government support or ecosystem services can help mitigate extreme negative outcomes.

However the impacts of extreme weather events on life satisfaction appear to be context specific. Gunby and Coupé (2023) analyse weather-related home damage in Australia and find that while such events have negative effects on subjective well-being, these effects tend to be modest and often statistically insignificant​. This conclusion is in contrast to the aforementioned studies showing larger and more persistent life satisfaction impacts from extreme weather events.

In summary, these life satisfaction studies reinforce the argument that experienced utility provides a more comprehensive measure of climate risk impacts than traditional economic models. By incorporating well-being metrics alongside economic losses, policymakers can design better informed and targeted interventions, ensuring that risk mitigation strategies account for both immediate financial costs and longer-term psychological resilience.

#### Integrating Regret Minimization into Consumer Preferences for Environmental Goods

Beyond experienced utility, Kahneman’s exploration of alternative decision theories has encouraged environmental economists to consider other psychological drivers, such as regret minimization, in climate-related choices. Piracci et al. (2023) apply Random Regret Minimization to consumer choices specifically for sustainable packaging options in the food sector, including bioplastic packaging and unpackaged (loose) food items. Their study reveals that anticipated regret, rather than utility maximization, underpins a substantial portion of consumer preferences, particularly in the context of reducing greenhouse gas emissions through more sustainable packaging. Many consumers choose sustainable packaging not merely to maximize utility but to minimize the regret they might feel if they did not make an environmentally responsible choice. This insight highlights the role of regret minimization as a driver of pro-environmental behavior, where individuals’ priorities avoiding future regret associated with environmentally harmful choices.

This finding underscores the role of regret as a powerful motivator, often overshadowed by traditional utility-driven models. For policymakers and businesses, this insight suggests that marketing sustainable products with messaging that highlights the potential regret of missing out on eco-friendly choices could increase adoption. Thus, regret minimization opens a new pathway for encouraging sustainable consumer behavior, showing how decision theories aligned with Kahneman’s insights can reveal motivational drivers that extend beyond simple utility.

#### Climate Shocks, Risk Preferences, and Rank-Dependent Utility

Kahneman’s influence also extends to studies of risk preferences under extreme environmental stress, particularly in contexts where Rank-Dependent Utility Theory offers a richer understanding of behavior than Expected Utility Theory alone. Holden and Tilahun’s (2024) study in Ethiopia, for example, finds that exposure to droughts increases risk-taking among young adults, a finding that contradicts traditional Expected Utility Theory predictions of increased risk aversion. Here, Rank-Dependent Utility Theory, combined with Kahneman’s insights into diminishing sensitivity and probability weighting, helps explain why individuals in high-stakes environments exhibit risk-seeking behavior under conditions of significant loss.

This behavior can be understood as an adaptive response, where high-risk choices are made to secure potential gains that could offset the extreme losses caused by drought. By showing that risk preferences are not fixed but instead fluctuate in response to environmental stress, Holden and Tilahun’s research highlights the limitations of Expected Utility Theory in predicting real-world behaviors under climate risks. Their findings emphasize the need for adaptive policies that consider individuals’ increased risk tolerance under stress, such as targeted financial support or risk mitigation tools in drought-prone areas. Together, this work illustrates how behavioral insights inspired by Kahneman can improve policies by accounting for the psychological adjustments people make in response to adverse conditions.

#### Bridging Decision and Experienced Utility: Implications for Climate Risk Policy

The studies discussed above bridge Kahneman’s concepts of experienced utility and decision-making under uncertainty, highlighting the relevance of subjective well-being data, regret minimization, and adaptive risk preferences for climate policy. Each approach contributes unique insights into how people perceive, adapt to, and mitigate climate risks. Experienced utility metrics provide an empirical foundation for assessing non-economic costs of environmental shocks, as seen in the research by Jones (2023), Luechinger and Raschky (2008), and Hudson et al. (2019) on dust storms and floods. These studies collectively suggest that traditional economic assessments may underestimate the true costs of climate risks by failing to capture losses in well-being.

The studies on regret minimization and Rank-Dependent Utility Theory further expand on Kahneman’s influence by incorporating non-standard motivations into consumer choices and risk-taking behaviors under climate stress. Piracci et al. (2023) reveal that anticipated regret, rather than utility maximization alone, influences consumer preferences for sustainable goods. This insight suggests that companies and policymakers could encourage sustainable behavior by emphasizing the psychological rewards of environmentally friendly choices. Meanwhile, Holden and Tilahun’s (2024) work on Rank-Dependent Utility Theory shows that risk preferences shift in high-stakes situations, revealing a behavioral response that policymakers can use to tailor adaptation strategies for vulnerable populations.

#### Kahneman’s Legacy in Environmental Economics: Experienced Utility and Alternative Decision Theories in Climate Risk Contexts

Kahneman’s contributions to experienced utility and alternative decision theories continue to shape climate policy, offering a more holistic understanding of decision-making under environmental risks. By moving beyond utility maximization, these studies reveal how subjective well-being, regret minimization, and adaptive risk preferences drive decisions in response to climate change. Kahneman’s legacy provides the analytical tools to understand these complex responses, supporting climate adaptation policies that prioritize human well-being and recognize the psychological dimensions of climate resilience.

### Dual Process Theory

Kahneman’s Dual Process Theory, which distinguishes between fast, automatic System 1 and slow, reflective System 2 thinking, has provided environmental economists with a framework to understand how individuals make climate-related choices. Dual Process Theory suggests that decisions can be influenced without full cognitive deliberation, a principle employed by “nudges” designed to align immediate, intuitive decisions with long-term environmental goals. Environmental behavior interventions increasingly use nudging as a low-cost, low-effort approach, targeting System 1 to encourage sustainable choices.

#### System 1 Nudges for Promoting Sustainable Consumption

Kurz’s (2018) study on reducing meat consumption in university restaurants in Gothenburg demonstrates how Dual Process Theory can promote sustainable food choices through nudging, contributing to climate change mitigation by reducing food-related greenhouse gas emissions. By modifying the menu to make vegetarian options more visible and accessible, the intervention aimed to engage System 1, triggering automatic behavior changes without requiring deliberate decision-making. This approach aligns with Kahneman’s insight that small, seemingly inconspicuous environmental changes can significantly impact behavior when System 1 is engaged. The study found that positioning vegetarian options prominently on the menu increased vegetarian meal consumption by 6% points during the intervention and sustained a 4-percentage-point increase after the intervention ended. This shift not only reduced food-related greenhouse gas emissions by 5% but also demonstrated that nudges can have a lasting impact on behavior, even after the intervention’s removal. These results suggest that targeting System 1 can facilitate enduring shifts in behavior, a valuable insight for policymakers seeking cost-effective strategies to promote sustainable consumption. Similar findings have been found in other contexts such as in experiments that mimic online food delivery platforms (Banerjee et al. 2023a).

Another application of System 1 nudges is in the use of commitment devices. For example, Panzone et al. (2024) show that prompting individuals to make a commitment before selecting groceries in an online supermarket reduces their carbon footprint by 9–12%. Banerjee et al. (2023a) find that individuals who commit in advance tend to reduce their intended selection of meat- and dairy-based products, making more pro-environmental dietary choices. However, commitment-based nudges can sometimes backfire in the short term, particularly when individuals resist the imposed constraint (see Banerjee et al. 2023b).

Eco-labels represent another widely used System 1 nudge. These labels often follow a traffic-light scheme, categorizing products as red, amber, or green based on their carbon footprint (Banerjee et al. 2023a). The underlying mechanism is that individuals automatically associate these colors with traffic signals, where red implies stop, green means go, and amber suggests caution. Extensive experimental studies have tested eco-labels in various settings, including student cafeterias, where they have been shown to reduce the likelihood of selecting a high-carbon meal by 2.7% points (Lohmann et al., 2023).

Information experiments have gained prominence in economics (and particularly environmental economics) recently (Haaland et al. 2023). These experiments rely on making information more salient to receivers underlying the idea that that information provision can help close awareness gaps and/or tap into people’s salience bias, which suggests that people are more likely to act based on what is more salient to them at the point of decision-making, yet another example of the system 1 nudge. For example, a recent study by Clo et al. (2024) using 1000 households in Italy suggests that providing information via smart meters shows that social feedback reduced per capita water consumption by around 6% (25.8 L per day or 6.8 gallons in total).

Framing effects offer yet another application of System 1 nudges, leveraging individuals’ psychological biases related to gains, losses, and the status quo. Arana and León (2013), for instance, find that default opt-in mechanisms for carbon offset programs significantly increase contributions compared to opt-in schemes requiring active participation. By reframing choices in alignment with behavioral tendencies, such interventions can effectively encourage pro-environmental behavior.

In summary, we have discussed how System 1 nudges—interventions that leverage automatic and intuitive decision-making—can promote sustainable consumption, particularly in dietary choices. Overall, these findings suggest that well-designed System 1 nudges can create lasting behavioral shifts, offering policymakers cost-effective strategies to promote environmental sustainability.

#### Policy Implications of Dual Process Theory in Environmental Nudges

These findings highlight the potential of Dual Process Theory for designing climate interventions that bypass the need for conscious, System 2-driven deliberation. By making environmentally friendly options easier to choose using defaults and more salient using commitment devices, labelling, and framing, policymakers can encourage sustainable behaviors in public institutions and other settings without relying on complex incentive structures or mandates. This literature on the use of such System 1 nudges is growing over time, and while we are not exhaustive in this review, these examples show the power of these behaviorally-informed interventions inspired by dual process theories. This approach provides a promising path for climate policies that require minimal cognitive effort from individuals, promoting sustainability through intuitive, automatic responses.

## Discussion of Key Findings and their Relevance for Climate Policies

### Discussion of Key Findings About Individual Decisions Regarding Climate Risks

The results that follow from applications of Kahneman’s Prospect Theory suggest important insights on the effects of bounded rationality on individual preparedness for climate risks. The demand for public policies and individual measures to mitigate climate change risks depends on the complex interplay of individual perceptions of probabilities of climate-related losses, the weighting of probabilities in decisions, and loss aversion. For example, if individuals correctly perceive probabilities of climate related losses, then Botzen and van den Bergh (2009) showed that loss aversion and probability overweighting results in higher demand for protection against climate risk than predicted by traditional Expected Utility Theory. The importance of reference dependence and loss aversion in the climate context was confirmed by studies like Koetse and Brouwer (2015) and Sterner and Adamowicz (2019). For probability weighting, Glatt et al. (2019) observed that probability overweighting generally occurs for flood risk in Switzerland. In contrast, Robinson and Botzen (2020) illustrate that underweighting may occur in a very low-probability context of flooding in the Netherlands, due to the application of a threshold level of concern model.

This finding of probability underweighting may be explained by the rare experience of natural disasters. This finding aligns with studies that tested Kahneman’s availability heuristic, and showed it explains an underestimation and underweighting of low-probability climate risk. This is because individuals who have experienced a natural disaster in the past may find it easier to imagine that such a disaster happens again in the future, compared to those without such experiences. The empirical literature we reviewed universally confirmed this availability bias in perceptions of drought, typhoon, wildfire and flood risk that were elevated after experiencing such a disaster (Gallagher 2014; McCoy and Walsh 2018; Ji and Cobourn, 2020; Wolf and Takeuchi 2022; Lohmann and Kontoleon 2023). Other somewhat related evidence supports Kahneman’s representativeness heuristic, suggesting that individuals after experiencing a natural disaster are more likely to invest themselves in protection or appreciate government-provided protection (Volkman-Wise 2015; Wolf and Takeuchi 2022). The mechanism here is that individuals have elevated risk perceptions if they believe the disaster experience was representative for the risk they face or believe protection is effective for future disaster events if it effectively performed in the past. However, the literature on the availability bias also pointed out that the effects of disaster experiences are often temporary, as elevated risk perceptions after a disaster tend to fade over time (Gallagher 2014; McCoy and Walsh 2018; Hennighausen and Suter 2020).

### Discussion of Relevant Insights for Climate Policy

The potentially significant economic impacts of climate change, such as more severe natural disaster losses, have spurred extensive research on policies to reduce greenhouse gas emissions or mitigate the societal impacts of climate change. The importance of this research is further underlined by studies that were inspired by Kahneman’s work on experienced utility, since these studies revealed the large impacts that natural disasters have on individual’s well-being (Luechinger and Raschky, 2008; Jones 2023). Moreover, evidence has shown that climate adaptation measures can limit the reductions in well-being that individuals report after experiencing such disasters. Examples of these adaptation measures include insurance coverage, emergency response to early warnings, and structural adjustments to buildings to withstand the impacts of extreme weather (Luechinger and Raschky’s, 2008; Hudson et al. 2019; Jones 2023).

However, studies focused on individual bounded rationality and behavioral biases suggest that many individuals in areas that are prone to natural disasters do not take cost-effective measures that limit natural disaster risks (e.g., Kunreuther and Botzen 2022). This means they are not well-prepared for the impacts of climate change. Explanations for this behavior are that individuals underestimate low-probability climate risk and underweight this risk in disaster preparedness decisions. Research inspired by Kahneman’s availability and representativeness heuristics found that this behavior is especially likely to occur if people have not personally experienced a natural disaster for some time (e.g. Wolf and Takeuchi 2022; Lohmann and Kontoleon 2023). Thus, there is a need for behavioral public policies to address these biases and encourage individuals to take cost-effective risk reduction measures before disasters occur.

Improved risk communication strategies have been proposed to correct biases in individual perceptions of natural disaster risks and make people pay attention to low-probability disaster risk. An example is communicating low flood probabilities over longer time horizons, such as 30 years. Cumulative probabilities of experiencing a loss over this long time are more likely to exceed someone’s threshold level of concern than the low annual loss probability (Bradt 2022). Lessons for risk communication strategies can be drawn from findings of applications of Kahneman’s ideas, which found that framing effects influence individual valuations of climate risk, and hence WTP or support for climate risk reduction. For instance, the outrage effect implies individuals are more likely to support climate action when human causes are emphasized as the root of the problem. Reference dependence furthermore suggests that framing a climate-related risk, such as disease spreading due to climate change, as a new risk enhance demand for protection against the risk (Slunge et al. 2019). Regret minimization implies that stressing regret of being unprepared for disasters or regret of making a climate unfriendly consumption choice in communication messages can steer behavior towards decisions that mitigate climate risk (Piracci et al. 2023).

However, correcting biases in risk perceptions may not be easily done, given the evidence for the confirmation bias of Kahneman implying that there is a tendency to favor information that supports existing beliefs. This bias has also been observed in the climate change context (Zappalà 2023). Therefore, it may be more effective to combine risk communication strategies with other behavioral public policies. An example is a default nudge that works with the status quo bias by setting protection as default (Robinson et al. 2021). Natural disaster coverage can be included by default in insurance policies and natural disaster risk reduction measures can be integrated into standard construction packages for new homes. Individuals are less likely to opt out of these protection measures due to status quo bias and loss aversion. The reason is that evidence for loss aversion showed that people demand more compensation to forgo protection against climate risk than they are willing to pay to secure it (Koetse and Brouwer, 2015). Nudges could also be applied for stimulating climate actions that reduce greenhouse gas emissions, such as having energy saving measures in place as default. Another example is the System 1 thinking nudges, as proposed by Kurz’s (2018), which make climate-friendly options more visible to consumers, such as vegetarian options on a menu.

Furthermore, the systematic biases in individual decision-making processes about investments in climate risk reduction measures may be a reason for implementing stricter regulations and financial incentives to stimulate uptake of these measures. Regulations can focus on spatial planning policies that prohibit new construction in high-risk areas. Additional regulations entail minimum building code requirements in disaster-prone areas that make properties more resistant to the impacts of extreme weather (Aerts et al. 2014). Differentiating premiums of insurance policies that cover extreme weather risks has been proposed as an effective price signal of risk that may correct biased individual risk perceptions (Kunreuther 2021). Moreover, such insurance policies may reward risk reduction investments of policyholders by giving them a premium discount that reflects the reduced climate risk. Experimental studies have shown that such financial incentives from insurance effectively stimulate investments in climate risk reduction measures (Mol et al. 2020a, b). Combining regulations that set minimum standards with risk communication, nudges, and financial incentives to stimulate risk reduction measures beyond these standards is likely the most effective approach to enhance individual preparedness for climate risks.

## Conclusion

Kahneman’s work has been highly influential in understanding individual decision making under risk and uncertainty, and the associated bounded rationality and behavioral biases. Kahneman’s three notable research programs with Tversky, as self-summarized in his Nobel Prize acceptance speech, addressed the “heuristics that people use and the biases to which they are prone under uncertainty”, “prospect theory, a model of choice under risk”, and “framing effects and their implications for rational-agent models” (Kahneman 2003, p. 1449). Its applications span numerous disciplines and fields, including environmental economics and climate change, as exemplified by the studies reviewed in this paper. These strands of research are not only relevant today but are increasingly vital in environmental economics as we confront wicked problems such as climate change, biodiversity loss, and environmental pollution. Insights from these theories have supported—and continue to support—the justification, design, and delivery of various (environmental) regulatory tools, prominently behavioral nudges which are widely used today in (environmental) public policy (Banerjee and John 2023).

The applications we reviewed in this paper demonstrate that Kahneman’s decisions theories and ideas are largely confirmed in the climate change context. Regarding Prospect Theory, the various reviewed studies find important effects of reference dependence, loss aversion, and probability weighting in decision-making with respect to climate change risks. These effects are likely to be highly context specific, as for example the over- or underweighting of flood risk in different countries has showed (Glatt et al. 2019; Robinson and Botzen 2020). Moreover, systematic differences in probability weighting of climate-related risks have been observed between population subgroups (Robinson and Botzen 2020). Nevertheless, only a few studies examined this in detail, making it difficult to draw generalized conclusions. An important avenue for future research is to increase our understanding about in which contexts and among which population subgroups specific climate change risks are under- or overweighted in climate adaptation or mitigation decisions. It is likely that availability bias and the representativeness heuristic play a role here, which have been studied more extensively for climate risks. These reviewed studies consistently find that individuals after experiencing a natural disaster have elevated perceptions of the risks posed by this disaster type, which may translate into probability (over-)weighting.

Future research in this field can benefit from various methodological innovations. Most existing studies examine individual decision making under climate risk by collecting survey or experimental data at a single point in time. However, this fails to adequately capture dynamics in climate adaptation or mitigation decisions, for instance due to the availability bias in response to natural disaster occurrences. These dynamics can be better analyzed using panel data studies, for example, using repeated surveys of the same population over time (e.g. Botzen et al. 2024). Moreover, the intention-behavior gap implies that combing survey and experimental approaches with field experiments of actual behavior can be a fruitful way forward to better understand drivers of climate adaptation and mitigation in practice (Kesternich et al. 2025). Moreover, the application of Machine Learning techniques to large-scale behavioral studies on decision-making about climate risk can advance the further testing of Kahneman’s theories and ideas (Peterson et al. 2021).

Finally, it is notable that the reviewed studies focus on studying individual decision-making about a single natural hazard, mainly floods, while individuals may encounter multiple correlated natural hazard risk at the same location, such as floods, storms and extreme heat. It is pertinent that future research examines how Kahneman’s theories and ideas apply in a multi-hazard climate risk context.

We discussed various recommendations for policies that help individuals in better preparing for climate change risks, including risk communication strategies, nudges, and financial incentives. We think there is an important task for behavioral/environmental economists to systematically test the effectiveness of these policies in various contexts of climate change risks. Ideally this evidence base is strengthened using both laboratory and field experiments to identify effective policies and combinations thereof.

Beyond Kahneman’s extensive body of work, which matured during his lifetime, there remain several ideas he initiated but did not fully explore. These concepts are now resurfacing in behavioral and decision sciences and hold promise for innovative policy solutions in environmental economics. For instance, Kahneman’s intuition about reasonableness as a better metric for understanding human behavior (Banerjee and Dold 2025) offers an alternative to merely cataloging biases. It provides a more intuitive framework for analyzing why “people with knowledge of [certain] situation(s) (including social pressures) would be able to see the behaviors as a satisfactory way to achieve a particular goal” (Madsen et al. 2024, p. 583). This approach is particularly relevant for addressing challenges such as individuals underweighting losses and underinvesting in climate adaptation measures, such as insurance. It allows policymakers to adopt a pragmatic perspective on individuals’ “reasonable” goals over varying time horizons, rather than narrowly categorizing them as myopic or irrational agents.

We believe that the fruits of Kahneman’s insights will continue to flourish in the field of behavioral-environmental economics studies about climate change risks, ensuring that his ideas thrive timelessly and extend their influence well beyond his time.

## Footnotes

1 We collected literature for this study by first searching the journals Environmental and Resource Economics, Journal of Environmental Economics and Management, Resource and Energy Economics, and Land Economics with the following search terms: “Kahneman and climate change”, “Kahneman and natural disaster”, and separately “Kahneman”. The papers that emerged from this search were screened and are included in our review if they concerned decision-making with respect to risks associated with climate change, and were motivated by Kahneman’s research. Furthermore, we added relevant economic studies from other journals that were identified through cross-referencing or known by the authors. A limitation of our approach is its potentially limited comprehensiveness. Our study differs from other reviews on behavioral economic applications in the field of environmental economics (e.g., Hepburn et al. 2010; Gsottbauer and van den Bergh 2011; Folmer and Johansson-Stenman 2011; Croson and Treich 2014), by specifically focusing on analyzes of decision making with respect to climate risks that were motivated by ideas of Kahneman.

2 See Wakker (2010) for a comprehensive guide to Prospect Theory.
